# Characterization and Optimization of Vesicle Properties in bioPISA: from Size Distribution to Post‐Assembly Loading

**DOI:** 10.1002/adbi.202400483

**Published:** 2024-12-18

**Authors:** Andrea Belluati, Adrian Bloch, Kaloian Koynov, Mariana Müller Nieva, Mohadeseh Bagherabadi, Annette Andrieu‐Brunsen, Harald Kolmar, Nico Bruns

**Affiliations:** ^1^ Department of Pure and Applied Chemistry University of Strathclyde Thomas Graham Building, 295 Cathedral Street Glasgo G1 1XL UK; ^2^ Department of Chemistry Technical University of Darmstadt Peter‐Grünberg‐Straße 4 64287 Darmstadt Germany; ^3^ Centre for Synthetic Biology Technical University of Darmstadt Peter‐Grünberg‐Straße 4 64287 Darmstadt Germany; ^4^ Max Planck Institute for Polymer Research Ackermannweg 10 55128 Mainz Germany

**Keywords:** artificial cells, enzymatic polymerization, PISA, polymersomes

## Abstract

This study investigates the formation and properties of vesicles produced via biocatalytic Polymerization‐Induced Self‐Assembly (bioPISA) as artificial cells. Methods for achieving size uniformity, including gentle centrifugation and sucrose gradient centrifugation, are explored, and the effects of stirring speed on vesicle morphology is investigated. The internal structure of the vesicles, characterized by a polymer‐rich matrix, is analyzed using fluorescence correlation spectroscopy (FCS). Additionally, the feasibility of loading macromolecules into pre‐formed vesicles is demonstrated using electroporation, and a fluorescent protein as well as enzymes for a cascade reaction were sucesfully incorporated into the fully assembled polymersomes. These findings provide a foundation for developing enzyme‐synthesized polymeric vesicles with controlled morphologies for various applications, e.g., in synthetic biology.

## Graphical abstract

The paper explores the creation and characterization of vesicles through biocatalytic Polymerization‐Induced Self‐Assembly (bioPISA), focusing on achieving size uniformity using centrifugation techniques. It examines the effects of stirring speed on vesicle morphology and analyses the internal polymer‐rich structure using fluorescence correlation spectroscopy (FCS). Electroporation successfully loads fluorescent proteins and enzymes into pre‐formed vesicles, enabling cascade reactions and paving the way for advanced applications in synthetic biology.

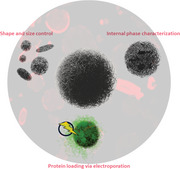

## 1 Introduction

The pursuit of mimicking life's formation processes offers valuable insights into understanding biological mechanisms and the origins of life. Moreover, it is essential for advancing our ability to manipulate biological systems and develop synthetic biology tools, namely artificial cells.^[^ ^1^ ^]^ Block copolymer vesicles, i.e., polymersomes, in particular, have garnered attention as artificial cell materials, due to their stability and chemical versatility. These synthetic vesicles can emulate cellular properties, including selective permeability, responsiveness to stimuli, and controlled degradation, making them ideal candidates for constructing robust and adaptable artificial cell models.^[^ ^2^ ^]^

A key technique in developing these biomimetic structures is Polymerization‐Induced Self‐Assembly (PISA).^[^ ^3^ ^]^ PISA involves the synthesis of amphiphilic block copolymers in aqueous environments, resulting in various structures like micelles and vesicles. This method is highly efficient at encapsulating biomolecules, making it an attractive option for creating cell‐like systems.^[^ ^4^ ^]^

Recognizing the potential of PISA, our group has investigated and developed a novel biocatalytic PISA (bioPISA), which uses enzymes (e.g., myoglobin, Mb) to initiate and control radical polymerizations that generate amphiphilic block copolymers which self‐assemble into vesicles during the polymerization reaction.^[^ ^5^ ^]^ This method uses biocompatible conditions, enhancing the process's compatibility with biological molecules and facilitating the encapsulation of these molecules under mild conditions. The ability to create more complex and functional structures through bioPISA marks a significant advancement in bottom‐up synthetic biology. Our initial study had already revealed a high degree of heterogeneity in the size and shape of the vesicles,^[^ ^5a^ ^]^ prompting further examination into this phenomenon, as size and shape are recognized to be important parameters in how vesicles, both nano‐ and microsized, interact with their environment.^[^ ^6^ ^]^

Here, we focus on understanding and optimizing vesicle properties using bioPISA. Motivated by the need to develop more efficient and functional artificial cells, we investigated both the external morphology and the internal composition of these vesicles. The size and shape of vesicles are critical parameters that determine their interaction with the environment,^[^ ^6a^ ^]^ and we observed that sampling the vesicle suspension at different heights yielded distinct size populations. This vertical size distribution suggested that vesicles of varying sizes settled or floated within the suspension medium. By refining our sampling techniques, we aimed to enhance the selectivity and uniformity of the vesicle populations, which was also possible via the alternative route of using different stirring speeds. This approach allowed us to produce vesicles with narrower size and shape distributions. In addition, we characterized the inner phase of the vesicles to better understand how the polymer‐rich interior influences their overall properties. Finally, we also explored the possibility of post‐assembly loading using electroporation, a technique that allows for the introduction of additional cargo into pre‐formed vesicles without disrupting their structural integrity. This dual focus on both the external and internal characteristics of the vesicles ensures they can be optimized for a variety of applications, enhancing their utility as functional artificial cells. Thus, we aim to provide a deeper understanding of how to optimize bioPISA vesicles for specific applications of novel hybrid systems that combine synthetic polymers with the complexity of natural molecules.

## 2 Results and Discussion

**Figure 1:**
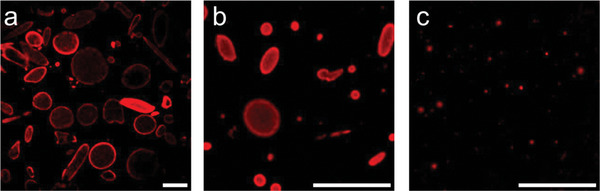
CLSM micrographs of bioPISA vesicles, sampled from a) the bottom section, b) the middle section, and c) the top section of a vesicle suspension. Red: CPC membrane label. Scalebars: 100 µm.

Vesicles were produced by grafting 2‐hydroxypropyl methacrylate (HPMA) on a poly(ethylene glycol) methyl ether (mPEG) ATRP macroinitiator (characterized by NMR and ATR‐FTIR spectroscopy, Figures S1 and S2, Supporting Information) using myoglobin, obtaining mPEG‐*b*‐PHPMA polymers that self‐assembled in vesicles, with a monomer conversion of 97%, in line with our previous report.^[^ ^5a^ ^]^ The observed prolate vesicle shape is likely due to several factors, including moderate shear forces during polymerization, variations in polymer concentration and chain length, kinetic trapping effects, and curvature influences from PEG orientation at the water interface.^[^ ^7^ ^]^ These conditions can favor elongated morphologies rather than perfectly spherical forms in PISA systems. In the original experimental setup, vesicles would be diluted 10× in the buffer in a 20 mL scintillation vial, and sampled from roughly the middle of the vial after quick mixing, then imaged by labeling their membrane with the probe Cy5‐PEG‐cholesterol (CPC).^[^ ^8^ ^]^ However, we observed via confocal laser scanning microscopy (CLSM) that sampling the vesicle mixture at different heights in 15 mL Falcon tubes yielded distinct shape and size populations: vesicles from the bottom appeared very large (>100 µm in diameter) (**Figure** **1**a) and slightly elongated: from the middle section (at ≈10 mL), vesicles were smaller, mainly ≈10–30 µm in diameter, with some smaller ones also present, and tendentially more elongated (Figure 1b). At the very top, vesicles were way below 10 µm in diameter and barely sedimented on the bottom of the chamber slide. This vertical size distribution suggested that vesicles of varying sizes settled or floated within the suspension medium and prompted us to further improve the spatial separation of these populations.

### 2.1 Characterization of the Vertical Vesicle Shape and Size Gradient

**Figure 2:**
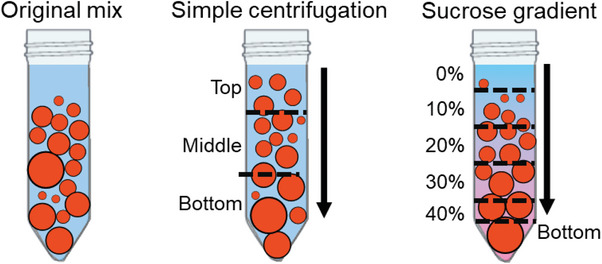
Sampling strategies of bioPISA vesicles, without centrifugation (original mix), simple centrifugation, and sucrose gradient centrifugation.

To characterize the vesicles produced by bioPISA, we further diluted the original mix (4 mL in 8 mL PBS) inside a 15 mL Falcon tube, in order to further increase the vertical resolution. We then centrifuged it at a slow speed (50 RCF, 5 min) and, via CLSM, remarked no evident change in the shape of the vesicles once fully resuspended (Figure S3, Supporting Information). This demonstrates that mild centrifugation could be applied to select our vesicles by size. Thus, we chose to compare vesicles after thorough mixing as the benchmark, vesicles after simple centrifugation (three different fractions: top, middle, and bottom), and vesicles centrifuged with a step sucrose gradient, from 0 to 40 w/v% (**Figure** **2**).

#### 2.1.1 Simple Centrifugation

Due to the size heterogeneity of the vesicles, many would not have their mid‐section on the same confocal plane, thus we used z‐stack 2D projections of samples in order to have a full representation. Additionally, as many vesicles had a non‐circular shape, the vesicle size was characterized by the surface area calculated for each object.

**Figure 3:**
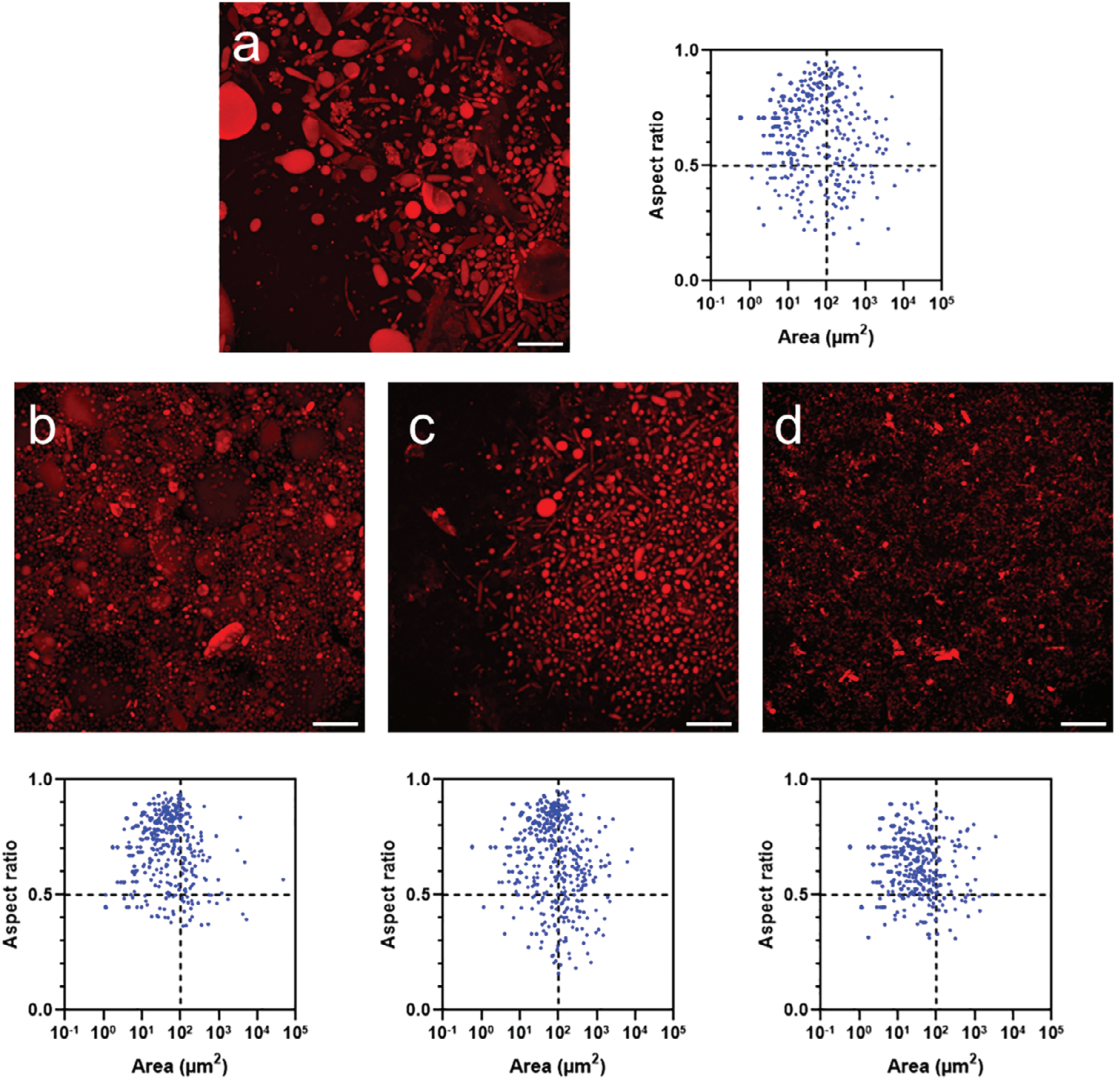
Z‐stack projections and analyses of various bioPISA vesicle populations. a) Original mix. b) Simple centrifugation, bottom fraction. c) Simple centrifugation, middle fraction. d) Simple centrifugation, top fraction. *N* = 500. The additional line at 100 µm^2^ indicates an arbitrary delimitation between smaller and larger vesicles; the line at aspect ratio 0.5 indicates an arbitrary delimitation between circular and more elongated shapes. Red: CPC membrane label. Scale bars = 100 µm.

The original mix, sampled at its middle level, showed a broad size distribution, with very large and very small vesicles (1–10 000 µm^2^) (**Figure** **3**a). The aspect ratio, expressed as the ratio between minimum and maximum diameters, was broadly distributed as well, with larger vesicles tendentially being less round. Summarizing both parameters (Table S1, Supporting Information), we could remark a median area of 17 µm^2^, a mean of 560 µm^2^ (coefficient of variation CV: 1024%), and a skewness of 21, indicating a strongly right‐tailed distribution, whereas the aspect ratio has a mean of 0.64 and CV 26%. With simple centrifugation and fractionation, we can observe the mean decreasing to 208 µm^2^, with CV 1027%, and skewness 21, for the bottom fraction (Figure 3b; Table S1, Supporting Information). The middle fraction has a similar (197 µm^2^) and much lower CV, 281%, whereas the top fraction has a mean of 77 µm^2^ and CV 334%, indicating generally smaller but, most importantly, more uniformly sized vesicles (Figure 3c,d; Table S1, Supporting Information). In all cases, the mean aspect ratio did not go above 0.71.

#### 2.1.2 Sucrose Gradient

**Figure 4:**
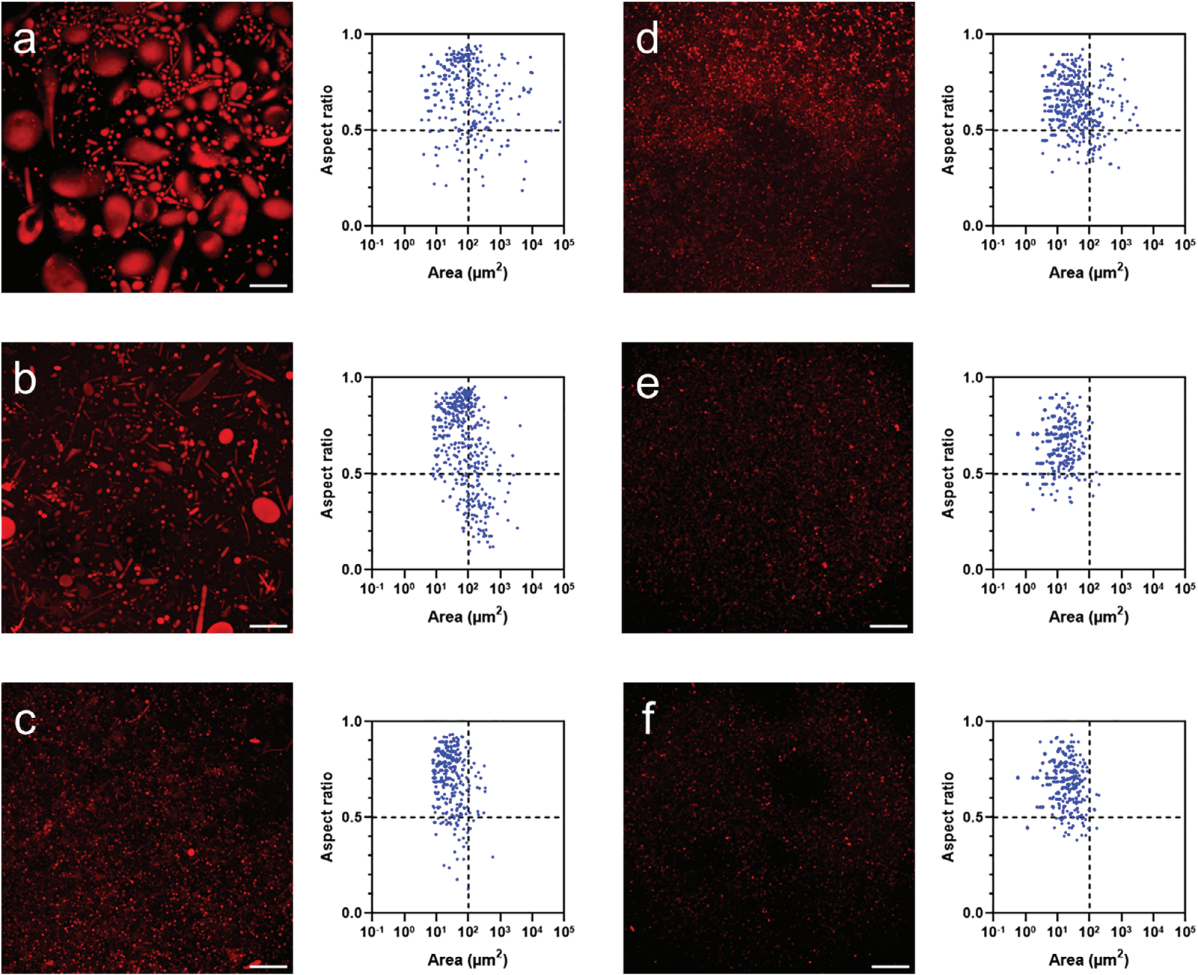
Z‐stack projections and analyses of various bioPISA vesicle populations after sucrose gradient centrifugation. a) Bottom fraction. b) 40% sucrose. c) 30% sucrose. d) 20% sucrose. e) 10% sucrose. f) 0% sucrose. *N* = 500, except for a) *N* = 326. The additional line at 100 µm^2^ indicates an arbitrary delimitation between smaller and larger vesicles; the line at aspect ratio 0.5 indicates an arbitrary delimitation between circular and more elongated shapes. Red: CPC membrane label. Scale bars = 100 µm.

When the vesicles were fractionated using sucrose gradient centrifugation, CLSM analysis showed that the vesicles distributed along the gradient with a sharper size distribution compared to the original and simple centrifugation methods. At the very bottom, sinking even in 40% sucrose, the mean size of the vesicles was 809 µm^2^ with a CV of 586% (**Figure** **4**a; Table S2, Supporting Information). In 40% sucrose, the mean size dropped to 146 µm^2^ and tended to decrease with the sucrose concentration (Figure 4b; Table S2, Supporting Information). Below 20%, only very small vesicles could be detected (Figure 4c–f; Table S2, Supporting Information). The mean aspect ratio did not increase dramatically, staying between 0.6 and 0.7, indicating that several non‐spherical structures are still present. Summarizing, the sucrose gradient effectively separated the vesicles into more distinct‐sized populations, with smaller vesicles concentrated in the lower‐density regions and larger vesicles in the higher‐density regions.

#### 2.1.3 Concentration of Vesicles

We then proceeded to quantify the vesicles per fraction to understand which populations tended to be produced the most via bioPISA. Using flow cytometry, we estimated ≈1.8 × 10^9^ vesicles in the original mix, i.e., 9 × 10^8^ vesicles mL^−1^. This remarkably high concentration is obtained thanks to the high polymer concentration of bioPISA. In contrast, other techniques can achieve, at most, 10^7^ vesicles mL^−1^ (via shaking^[^ ^9^ ^]^), whereas microfluidic vesicle formation can reach 10^5^–10^6^ vesicles mL^−1^.^[^ ^10^ ^]^

**Figure 5:**
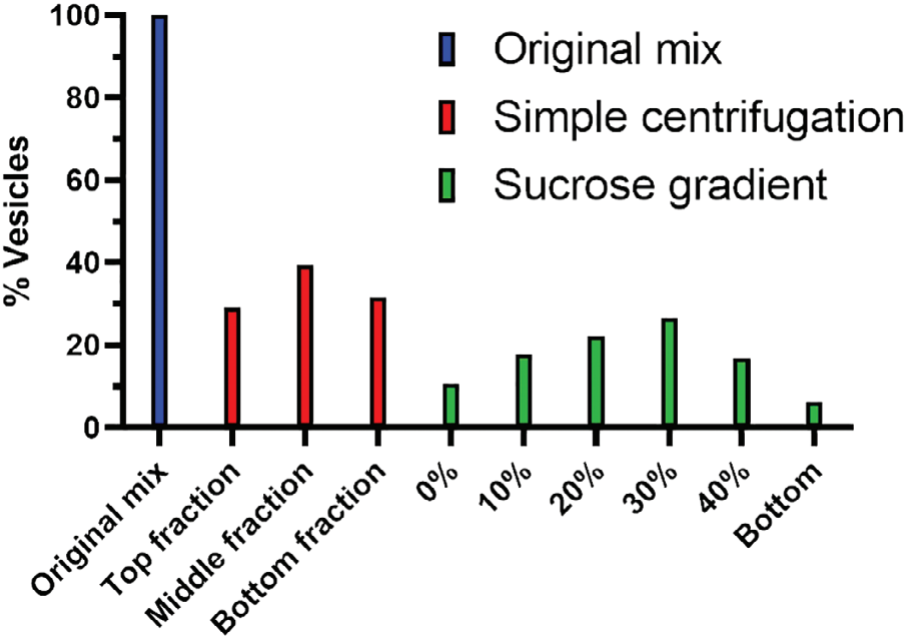
Relative population of bioPISA vesicles separated via simple centrifugation or sucrose gradient centrifugation.

The fractions derived from simple centrifugation were then measured, showing that 40% of the vesicles were in the middle phase, with the others evenly distributed between the top and bottom (**Figure** **5**). Similarly, with sucrose, the majority of the vesicles fell between 20 and 40%. These results show that bioPISA produces mainly slightly elongated vesicles with mid‐section surfaces roughly between 20 and 150 µm^2^ which, assuming a perfectly spherical object, means diameters between 5 and 14 µm.

### 2.2 Impact of Stirring Speed on Vesicle Formation

**Figure 6:**
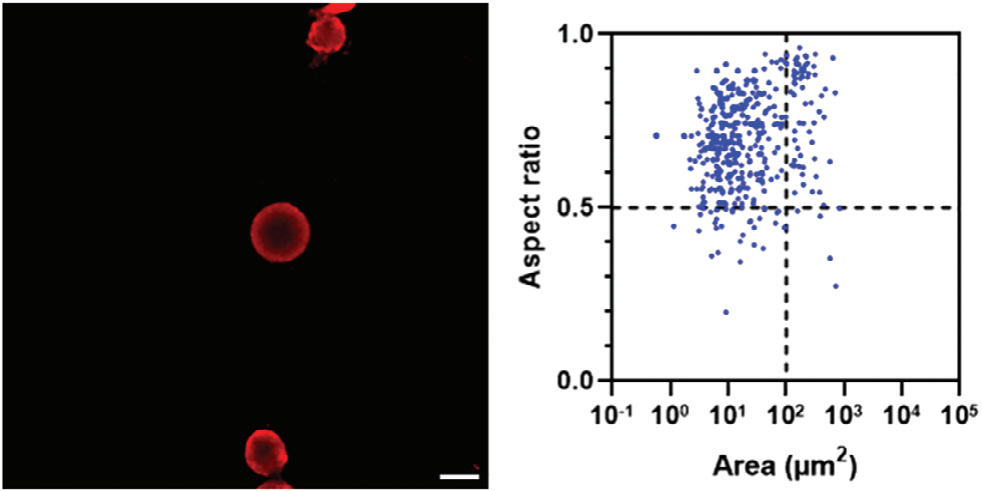
Z‐stack projection and analyses of vesicles produced with slow stirring during bioPISA. *N* = 500. The additional line at 100 µm^2^ indicates an arbitrary delimitation between smaller and larger vesicles; the line at aspect ratio 0.5 indicates an arbitrary delimitation between circular and more elongated shapes. Red: CPC membrane label. Scale bar = 20 µm.

As the size and shape of vesicles could also be affected by the stirring speed during bioPISA,^[^ ^11^ ^]^ we investigated its impact on the process. Slow stirring at 50 rpm resulted in the formation of smaller and more monodisperse vesicles (**Figure** **6**; Table S3 and Figure S4, Supporting Information), with a mean size of 50 µm^2^. In contrast, fast stirring at 400 rpm produced vesicles with irregular shapes and sizes, indicating that excessive agitation negatively affected vesicle formation, leading to poorly defined structures (Figure S5, Supporting Information).

### 2.3 Characterization of Vesicle Lumen

**Figure 7:**
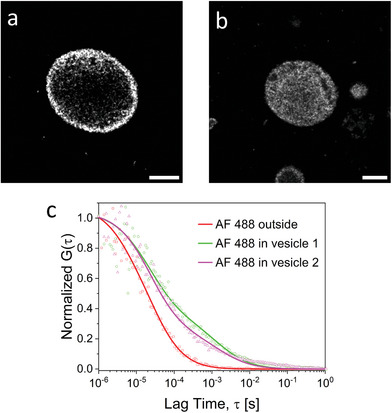
Characterization of the internal phase of bioPISA vesicles. a) CLSM micrograph (in reflection mode) of a “hollow” vesicle (vesicle 1). b) CLSM micrograph of a “filled” vesicle (vesicle 2). c) Normalized FCS autocorrelation curves of AF 488 diffusing outside and inside vesicles. The solid lines represent the corresponding fits with Equation 2. Scale bars: 5 µm.

During the characterization of the vesicls' size and shape, we noticed that CPC would not only stain the membrane but would also partially diffuse into the lumen and stain relatively well‐defined internal structures. In our previous study, we had already observed that 10%–20% of the vesicles had a polymer‐rich internal phase (Figure S6, Supporting Information),^[^ ^5a^ ^]^ and that the internal fluorescence was unlikely to derive from an internal partition of the probe, as it had not been observed with Pluronic L121 vesicles.^[^ ^12^ ^]^ Observing the vesicles via CLSM in reflection mode, with no fluorescent probe, yielded the same results: some of the vesicles appeared essentially empty (**Figure** **7**a; Figure S7a, Supporting Information), and others showed a dense polymer filling (Figure 7b; Figure S7, Supporting Information). To characterize the internal phase of this system, we employed fluorescence correlation spectroscopy (FCS), which allows us to probe the density of a macromolecular phase by measuring the diffusion time of a fluorescent molecule, which would increase by the interactions of the fluorphore with the polymer within the vesicle.^[^ ^13^ ^]^ As a probe molecule, we used Alexa Fluor 488 (AF 488, 643 Da) to diffuse into the vesicles, and measured its diffusion time (reverse proportional to its diffusion coefficient, see Experimental Section) outside and inside two vesicles (Figure 7c): vesicle 1 (no dense polymer phase) and vesicle 2 (dense polymer phase). AF 488 outside of the vesicles had an average diffusion time of 24 ± 2 µs, which is identical to that measured in reference experiments without any vesicles. Inside both kinds of vesicles, we observed more complex, two‐component diffusion behavior and the corresponding autocorrelation curves (Figure 7c) have to be fitted using Equation 2 with m = 2. For both kinds of vesicles, the first, fast diffusing population has a fraction f1 ≈80% and a diffusion time tD1 ≈60 µs. This value is ≈2.5 times bigger than the diffusion time of the AF 488 tracer in pure water. As discussed in earlier studies^[^ ^13^, ^14^ ^]^ such crowding‐induced slowdown is caused by excluded volume interactions, which only depend on the polymer concentration. The 2.5 fold slowdown suggests a polymer concentration of ≈15–20 w/v%.^[^ ^13^, ^14^ ^]^

The diffusion time *τ*_D2_ of the second, slow diffusing AF488 population was ≈4000 µs in vesicle 1 and ≈13 000 µs in vesicle 2. These much lower diffusion times originate from tracers that temporarily adsorb to the polymer chains and consequently either diffuse together with them^[^ ^13a^ ^]^ or become temporarily immobile (until desorption) if the polymer chains are two crowded or crosslinked and cannot diffuse on the length scale of the FCS observation volume (≈300 nm).^[^ ^14^ ^]^

In summary, a dense polymer interior in both types of vesicles notably retard the diffusion of the AF 488 probe through excluded volume interactions and transient adsorption onto polymer chains. This underscores the significant impact of internal polymer structure on probe mobility within vesicular systems.

In light of these results, we propose that our previously‐named giant unilamellar vesicles (GUVs) should rather be simply called Giant Vesicles (GVs) to account for the internal complexity, not necessarily resulting in a single lamella, but neither in easily discernible multiple ones.

### 2.4 Electroporation for Loading Macromolecules

The densely packed polymer lumen is a result of the self‐assembly process and can be seen as a loading of the GV with excess polymer. Similarly, until now, in bioPISA, macromolecules have been loaded directly during the self‐assembly process. However, encapsulation of macromolecules at this stage limits flexibility and control over the loading process. Recognizing this limitation, we explored electroporation as a method for loading macromolecules into pre‐formed vesicles, allowing control over the timing and quantity of the encapsulation, which was already successfully applied to nanometre‐sized polymersomes^[^ ^15^ ^]^ and lipid vesicles.^[^ ^16^ ^]^

**Figure 8:**
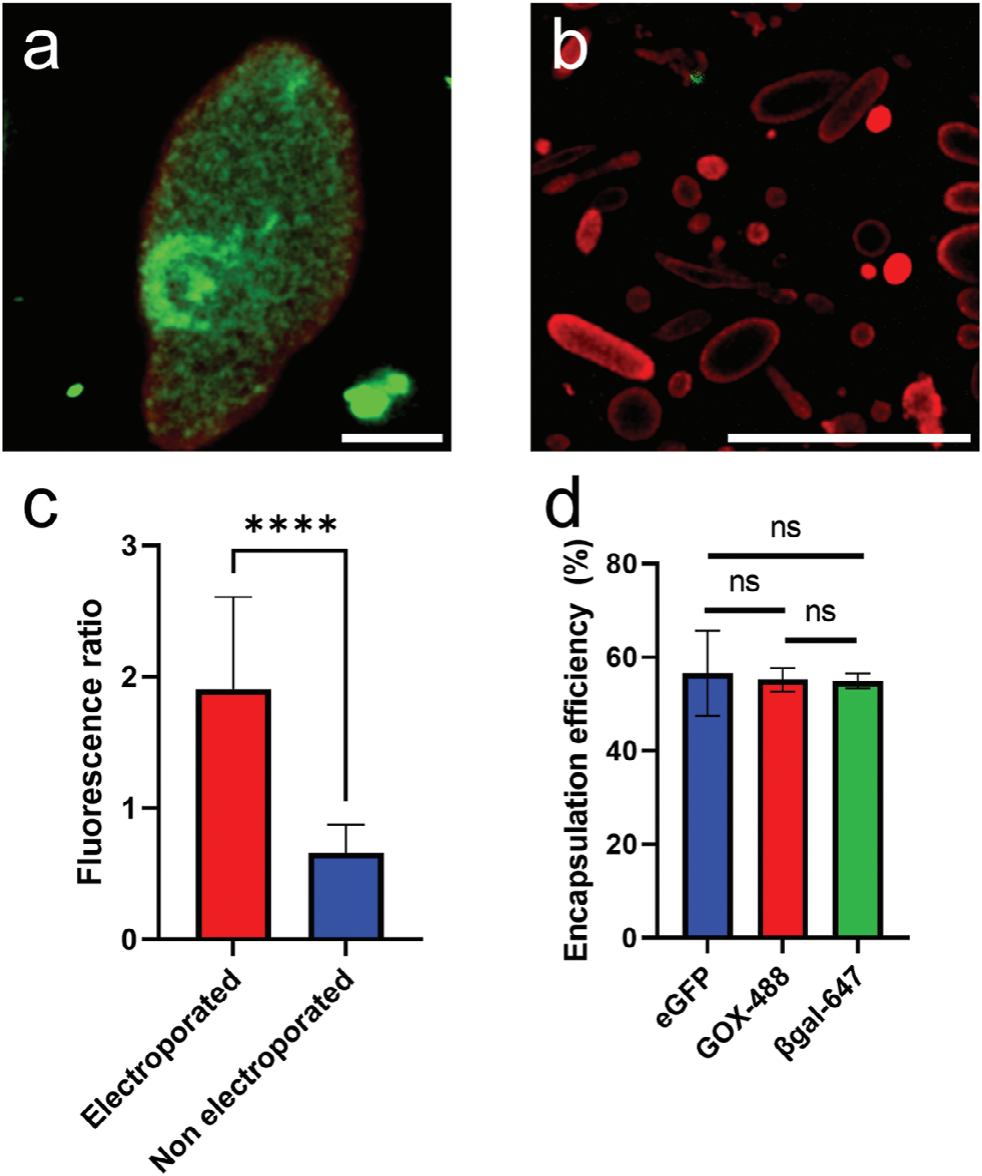
Electroporation of bioPISA vesicles to load them with eGFP and enzymes. a) CLSM micrograph of an electroporated vesicle with eGFP; scale bar: 5 µm. b) CLSM micrograph of non‐electroporated vesicles, scale bar: 100 µm. c) Fluorescence ratios (inside:outside) of electroporated eGFP‐loaded vesicles and of non‐electroporated vesicles. *n* = 15 vesicles, ± SD. ^****^: *p* < 0.0001 after Welch's *t*‐test. d) Encapsulation efficiency of eGFP and fluorescently labelled enzymes, *n* = 3 replicates, ± SD (one‐way ANOVA, p > 0.9). Some of the eGFP can be seen agglomerated outside of the vesicles, both with and without electroporation.

We successfully loaded the model protein enhanced green fluorescent protein (eGFP) into the GVs via electroporation and then washed them. CLSM confirmed the presence of eGFP inside the vesicles (**Figure** **8**a,b; Figure S8 and Table S4, Supporting Information), and the significantly different ratio between fluorescence inside and outside the vesicles demonstrated that it is possible to introduce large molecules into pre‐formed vesicles without compromising the vesicles' structural integrity. We could observe, in several electroporated vesicles, regions of higher fluorescence, further suggesting that the internal polymer produces discrete compartments, in which the protein tends to concentrate (Figure 8c), but without any discernible influence on encapsulation (Table S5, Supporting Information). We calculated the encapsulation efficiency to be above 50% (Figure 8d). Similarly, fluorescently labeled enzymes such as glucose oxidase (GOX‐488) and β‐galactosidase (βgal‐647) could be encapsulated at the same time with high efficiency. No difference in encapsulation efficiency was observed, and no preferential encapsulation could be observed for “filled” or “hollow” vesicles. Moreover, myoglobin that got encapsulated during bioPISA appeared not to leach out of the vesicles during the electroporation process, possibly due to the polymer‐dense lumen (Figure S9, Supporting Information).

Finally, non‐fluorescent enzymes were successfully co‐encapsulated, enabling a cascade reaction inside of the vesicles where lactose is converted to glucose and galactose by β‐galactosidase, glucose oxidase produces hydrogen peroxidase by glucose oxidation, and the peroxidase activity of myoglobin converts 3,3',5,5'‐tetramethylbenzidine (TMB) into a colored product.^[^ ^17^ ^]^ The colored product only formed when both enzymes (β‐galactosidase and glucose oxidase) had been electroporated into the vesicles. Otherwise, the washing step removed the enzymes from the GV suspension (Figure S10, Supporting Information). This demonstrates the functional versatility of the vesicles in hosting enzymatic cascade reactions. In further investigations we will study the loading of multiple biomacromolecules, aiming, for instance, at highly concentrated cell‐free expression systems within the vesicle.

## 3 Conclusion

This study provides an in‐depth analysis of the factors influencing the formation and properties of vesicles produced via bioPISA. We systematically investigated the effects of gentle centrifugation and sucrose gradient centrifugation, revealing these methods as effective for achieving size separation among vesicles and size selection of the vesicles. Furthermore, we demonstrated that varying the stirring speed during vesicle formation significantly impacts the vesicle morphology. Besides the outer membrane, we could also characterize and modify the internal phase of the vesicles: FCS revealed the densely‐packed lumen of vesicles, which could then be further loaded post‐assembly via electroporation with eGFP and enzymes.

In a broader context, these findings highlight the potential of bioPISA as a versatile platform for creating complex vesicular systems that host enzymatic cascade reactions. The ability to manipulate vesicle size, shape, and content opens new avenues for the design of enzyme‐synthesized synthetic biological systems that can emulate natural cellular processes and structures. Therefore, this work lays the foundation for the optimization of vesicle characteristics, supporting future developments in hierarchically structured and size‐controlled synthetic cell systems.

## 4 Experimental Section

### Materials

Wild‐type eGFP was produced according to a published protocol.^[^ ^18^ ^]^ Unless otherwise specified, all compounds were supplied by Sigma–Aldrich and used without further purification. Cy5‐PEG‐Cholesterol (CPC) was synthesized using cholesterol‐PEG4‐N_3_ according to the published protocol.^[^ ^12^ ^]^

### Synthesis of the mPEG Macroinitiator (mPEG‐BIB)

Requiring a bromoacyl moiety on a PEG to obtain an ATRP macroinitiator, mPEG‐bromoisobutyrate was produced using a modified protocol.^[^ ^5a^ ^]^ 182 µL of triethylamine (TEA) and 161 µL of 2‐bromoisobutyryl bromide (BIB) were dissolved in 25 mL dichloromethane (DCM). The solution was deoxygenated with Ar for 30 min and brought to 0 °C in an ice bath. Similarly, 5 g of methyl ether PEG (mPEG, average molecular weight 5000 Da) were separately dissolved in 25 mL DCM, deoxygenated, and cooled down. The mPEG solution was added dropwise to the TEA + BIB solution, left to react for 1 h at 0 °C, and then for 16 h at RT. The compound was then precipitated in ice‐cold diethyl ether, and centrifuged at 4000 RCF, 0 °C for 10 min. The solvent was discarded, and the obtained solid was dried under a vacuum for 2 days. The final mass yield was 72%. The compound was characterized via NMR (Varian Unity 300 MHz spectrometer) and ATR‐FTIR (Bruker Alpha II) spectroscopy. ^1^H NMR (300 MHz, CDCl_3_, δ): 1.92 (s, 6H, CCH_3_), 3.36 (s, 3H, OCH_3_), 3.40 (t, 2H, OCH_2_), 3.63 (OCH_2_), 4.31 (t, 2H, OCH_2_).

### bioPISA Procedure

In a typical bioPISA procedure, HPMA (2 mL, filtered on basic alumina) was measured in a 4 mL vial that was closed with a septum. 50 mg of mPEG‐BIB and 12 mg of sodium ascorbate (NaAsc) were dissolved in 700 µL PBS‐Br (PBS with 100 mM NaBr) buffer pH 7.4 spiked with 5 vol% DMSO in a 4 mL vial closed with a septum. 10 mg Mb was dissolved in 1.3 mL PBS‐Br in a 10 mL Schlenk flask. All the solutions were degassed with Ar for 30 min. The mPEG‐BiB/NaAsc solution was added to the Schlenk flask, and the resulting solution was stirred for 5 min. The color of the reaction mixture changed from brown to red (Mb reduction). Then, 400 µL of purified HPMA was added. The reaction mixture was stirred (typically 120 rpm, tested at 50 (slow stirring) and 400 rpm (fast stirring) as well) for 3 h at 37 °C before being opened to air to quench the polymerization by atmospheric oxygen. The final suspension was diluted with 18 mL of PBS.

### Sampling

4 mL of the suspension was further diluted in 8 mL PBS in a 15 mL Falcon tube.

For the original mix, the vesicle suspension was thoroughly mixed via vortex, and then sampled at the 7 mL mark of the Falcon tube.

For the simple centrifugation, the sample was centrifuged for 5 min at 50 x g (Megafuge 16R, Thermo Fisher). Then, fractions were collected until the 8 mL mark (top fraction), 4 mL (middle fraction), and the remaining suspension (bottom fraction).

For the sucrose gradient, sucrose was dissolved in PBS at 40, 30, 20, and 10 w/v%. 2 mL of each solution were carefully layered on top of one another in a 15 mL Falcon tube, then 4 mL of vesicle suspension were added on top of them and then quickly centrifuged at 50 x g. Afterward, fractions were collected, assigning the original sucrose concentration as the name of each (0% corresponding to the top 2 mL). At the bottom, a noticeable pellet of extra heavy vesicles could be isolated, which was resuspended in 0.6 mL of additional PBS.

### Imaging and Morphology Characterization via CLSM

10 µL of each sample were diluted in 200 µL PBS in Nunc Lab‐Tek 8‐well chamber slides (Thermo Fisher). The imaging was performed on a Leica SP8 CLSM, equipped with 20x and 63x water objectives (eGFP: ex. 488 nm, em. 505–525 nm; CPC: ex. 635 nm, em. 660–690 nm). Z‐stacks were taken and layered as 2D projections (average intensity) using ImageJ.^[^ ^19^ ^]^ The analysis of the vesicles was done using the particle analyzer function of ImageJ, and 500 random vesicles (when possible) were selected for analysis. The area was the area of the vesicles in their specific picture. The aspect ratio was defined as the ratio between minimum and maximum Feret diameter (i.e., the caliper diameters), where a non‐perfectly isometric figure has an aspect ratio <1.

### Vesicle Counting

Vesicles were further diluted 100 times in PBS and mixed 1:1 with Precision Count Beads (BioLegend) in order to have a reference sample for flow cytometry analysis, using a CytoFLEX S by Beckman Coulter (CytExpert Version 2.4.0.28). 50 000 events per sample were recorded. No gating was applied to the (unlabelled) vesicles in order to detect every event regardless of size, but the highly fluorescent cell counting beads were instead gated in order to determine their number. According to the supplier's protocol, the absolute vesicle count was determined with Equation 1.

(1) $$\begin{array}{cc} & \mathrm{Abso}\mathrm{lute}\;\mathrm{vesi}\mathrm{cle}\;\mathrm{count}\left(\frac{\mathrm{vesi}\mathrm{cle}}{\mu L}\right)=\frac{\mathrm{Vesi}\mathrm{cle}\;\mathrm{count}}{\mathrm{Prec}\mathrm{ision}\;\mathrm{Count}\;\mathrm{Beads}\;\mathrm{Count}}\\ & \;\times \;\mathrm{Prec}\mathrm{ision}\;\mathrm{Count}\;\mathrm{Beads}\;\mathrm{Concentration}\left(\frac{\mathrm{Beads}}{\mu L}\right) \end{array}$$

The total number of vesicles was then calculated backward from the known sample volume (measured with a micropipette) and its dilution factor.

As the bottom fraction from sucrose gradient centrifugation was strongly enriched with very large vesicles, it would clog the instrument's microfluidics. Instead, that fraction was measured by counting the vesicles via CLSM and extrapolating that to the total volume. Small discrepancies between the total amounts of vesicles from the different methods (5–10%) indicate that the method could provide an estimate of the number, which is sufficient for the order of magnitude of vesicle concentration.

### Vesicle Inner Phase Characterization – Imaging and FCS

The sample from the original mix was examined on an LSM 880 (Carl Zeiss). The excitation was done with the 488 nm line of an Argon laser focused into the studied samples through a C‐Apochromat 40×/1.2 W water immersion objective (Carl Zeiss). The emission light was collected with the same objective and after passing through a confocal pinhole, directed to a spectral detection unit (Quasar, Carl Zeiss) in which a detection range of 500–550 nm was selected. Eight‐well polystyrene chambered cover glasses (Nunc Lab‐Tek, Thermo Fisher Scientific) were used as sample cells for the studied solutions. The vesicles were first imaged using reflection mode, which does not require labeling if the material is dense enough. Then, the confocal volume was positioned in the center of a vesicle or in the space between the vesicles and a series of 15 FCS measurements with a total duration of 150 s were performed. The time‐dependent fluctuations of the fluorescent intensity δF(τ) were recorded and analyzed by an autocorrelation function G(τ) = 1 + 〈δF(t)∙δF(t + τ)〉/〈F(t)〉^2^. The obtained experimental autocorrelation curves were fitted with the following analytical expression:

(2) $$G\left(\tau \right)=1+\left[1+\frac{f_{T}}{1-f_{T}}e^{-\tau /\tau_{T}}\right]\frac{1}{N}\sum_{i=1}^{m}\frac{f_{i}}{\left[1+\frac{\tau }{\tau_{Di}}\right]\sqrt{1+\frac{\tau }{S^{2}\tau_{Di}}}}$$

Here, N is the average number of diffusing fluorescence species in the observation volume, f_T_, and τ_T_ are the fraction and the decay time of the triplet state, τ_Di_ is the diffusion time of the i‐th diffusion component, f_i_ is its fraction, and S is the so‐called structure parameter, S = z_0_/r_0_, where z_0_ and r_0_ represent the axial and radial dimensions of the confocal volume, respectively. Furthermore, the diffusion time, τ_Di_, is related to the respective diffusion coefficient, D_i_, through: $\tau_{Di}=\frac{r_{0}^{2}}{4D_{i}}$. The fits yielded the corresponding diffusion times, and subsequently the diffusion coefficients of the fluorescent species. As the value of r_0_ depends strongly on the specific characteristics of the optical setup, calibration experiments were performed using a fluorescent tracer with a known diffusion coefficient, i.e., Alexa Fluor 488 in water.

The experimental autocorrelation curves were fitted with Equation 2 using the software package ZEN Black (Carl Zeiss). For experiments performed outside of a vesicle, one‐component model (m = 1 in Equation 2) was used. For experiments performed in a vesicle, a two‐component model (m = 2 in Equation 2) was used.

### Vesicle Electroporation

Vesicles (from the original mix) were diluted 1:1 with a 4 mg mL^−1^ eGFP, a 4 mg mL^−1^ (Alexa Fluor 488)‐labeled GOX, or a 10 mg mL^−1^ (Alexa Fluor 647)‐labeled β‐gal solution in PBS (enzymes labeled according to a previous protocol^[^ ^5a^ ^]^). 100 µl of these solutions were placed in ice‐chilled 1 mm path electroporation cuvettes (VWR). The cuvette was inserted in an electroporator (Eppendorf 2510 electroporator) and subjected to 5 × 2500 mV pulses (mean time: 3 ms). The cuvette was then placed on ice for 3 h. Then, the content was diluted with 400 µL PBS, centrifuged two times at 5000 x g, 5 min, washing the pellet to remove as much unencapsulated protein as possible.

The fluorescence of eGFP, GOX‐488, and βgal‐647 in the supernatant was measured, before and after electroporation, with a Clariostar Plus plate reader, and the change in fluorescence was used as an indicator of encapsulation. The presence of Mb in the supernatant was measured with vesicles subjected to electroporation without additional cargo, measuring the absorbance at 280 nm with a NanoDrop One/OneC UV–vis spectrophotometer (Thermo Scientific).

The TMB cascade reaction assay was performed by mixing in a PMMA microcuvette: 50 µL of GV suspension, 50 µL of a 5 mg mL^−1^ TMB solution in ddH_2_O, and 100 µL of 300 mM lactose in PBS, finally diluting it to 1 mL with PBS. The cuvettes, in triplicate, were incubated at 37 °C for 30 min before their absorbance at 420 nm was measured using a Cary 60 UV–vis Spectrophotometer (Agilent).

### Statistical Analysis

Data was not pre‐processed for analysis, whereas image brightness and contrast were optimized for the figures. All data is presented as mean of *n* replicates, ± SD (see legends for n). Differences were tested between populations using two‐tailed *t*‐tests (or one‐way ANOVA for comparison of >2 populations), and *p*‐values are shown. For encapsulation studies with CLSM imaging, the proportions of “fluorescent” outcomes was compared between vesicle population groups using a two‐proportion *Z*‐test. The proportions were calculated as the count of “Yes” outcomes divided by the total counts for each group. Standard errors (SE) for each group's proportion were determined using the formula:

(3) $$SE=p\left(1-p\right)/n$$

where p is the proportion and n is the group size. A pooled SE was then computed to assess the difference in proportions, followed by a *Z*‐score and corresponding two‐tailed *p*‐value to test for statistical significance. GraphPad Prism v 9.0.0. was used for statistical analysis.

## Conflict of Interest

The authors declare no conflict of interest.

## Author Contributions

A.B. performed conceptualization, methodology, investigation, validation, data analysis, supervision, and funding acquisition. A.Bl. and K.K. performed the investigation and methodology. M.M.N. performed investigation. M.B. performed investigation, methodology, supervision, and visualization. A.A.B. and H.K. performed supervision. N.B. performed supervision, funding acquisition, and resources. All authors participated in the writing of the manuscript.

## Supporting information



Supporting Information

## Data Availability

The data that support the findings of this study are available from the corresponding author upon reasonable request.
