# Supplementary material for: Characterization and Optimization of Vesicle Properties in bioPISA: from Size Distribution to Post‐Assembly Loading
Source: Adv Biol (Weinh). 2024 Dec 18;9(5):2400483. doi: 10.1002/adbi.202400483 (PMC12078863; doi:10.1002/adbi.202400483)
Supplement: Supplementary file 1 — Supporting Information [file ADBI-9-2400483-s001.pdf]

ADVANCED BIOLOGY

Supporting Information

for *Adv. Biology*, DOI 10.1002/adbi.202400483

Characterization and Optimization of Vesicle Properties in bioPISA: from Size Distribution to Post-Assembly Loading

Andrea Belluati, Adrian Bloch, Kaloian Koynov, Mariana Müller Nieva, Mohadeseh Bagherabadi, Annette Andrieu-Brunsen, Harald Kolmar and Nico Bruns**

Supporting Information

Characterization and Optimization of Vesicle Properties in bioPISA: From Size Distribution to Post-Assembly Loading

Andrea Belluati, Adrian Bloch, Kaloian Koynov, Mariana Müller Nieva, Mohadeseh Bagherabadi, Annette Andrieu-Brunsen, Harald Kolmar, Nico Bruns**

A. Belluati, N. Bruns
Department of Pure and Applied Chemistry, University of Strathclyde, Thomas Graham Building, 295 Cathedral Street, Glasgow G1 1XL, UK
Centre for Synthetic Biology, Technical University of Darmstadt, Peter-Grünberg-Straße 4, 64287 Darmstadt, Germany
E-mail: andrea.belluati@tu-darmstadt.de nico.bruns@tu-darmstadt.de

A. Belluati, A. Bloch, M. Müller Nieva, M. Bagherabadi, A. Andrieu-Brunsen, H. Kolmar, N. Bruns
Department of Chemistry, Technical University of Darmstadt, Peter-Grünberg-Straße 4, 64287 Darmstadt, Germany

K. Koynov
Max Planck Institute for Polymer Research, Ackermannweg 10, 55128 Mainz, Germany

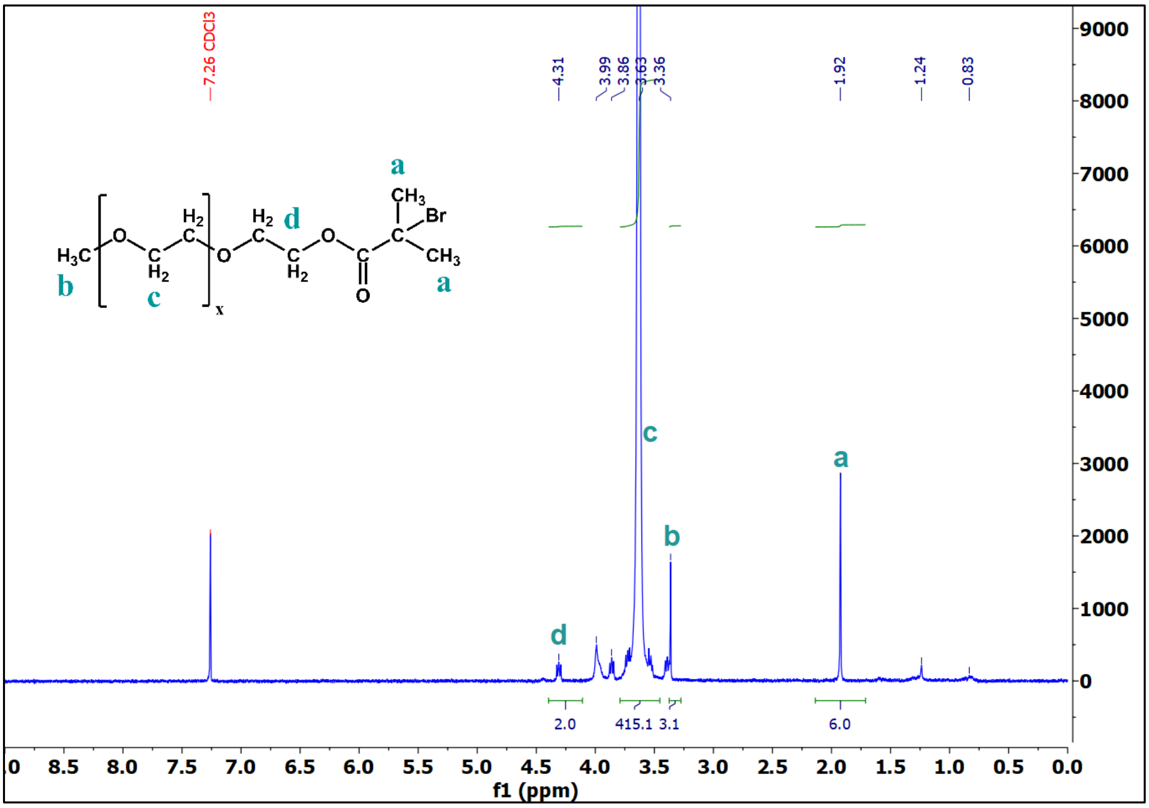

Figure S 1 ^{1}H-NMR in $CDCl_3$ (300 MHz) from mPEG macroinitiator (mPEG-BIB).

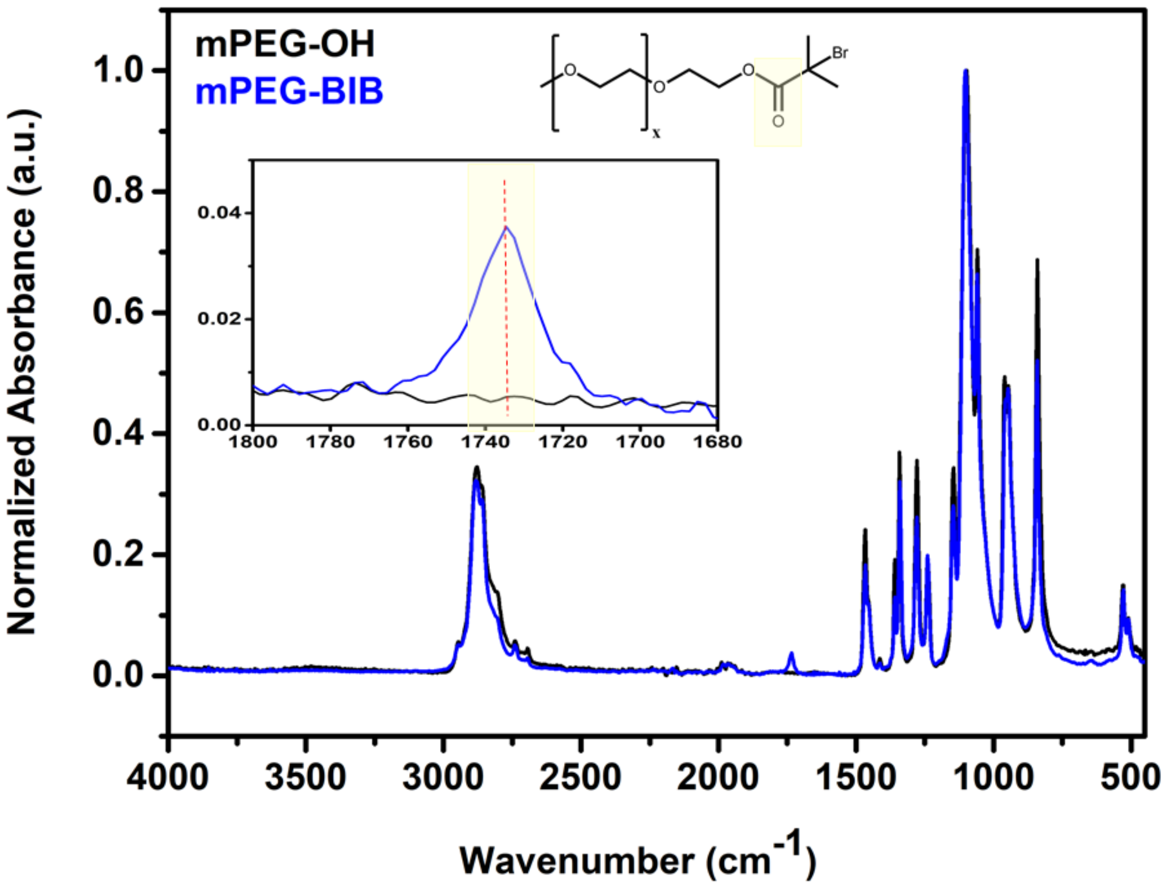

Figure S 2 ATR-FTIR Spectra from mPEG-OH (black) and mPEG macroinitiator (mPEG-BIB, blue).

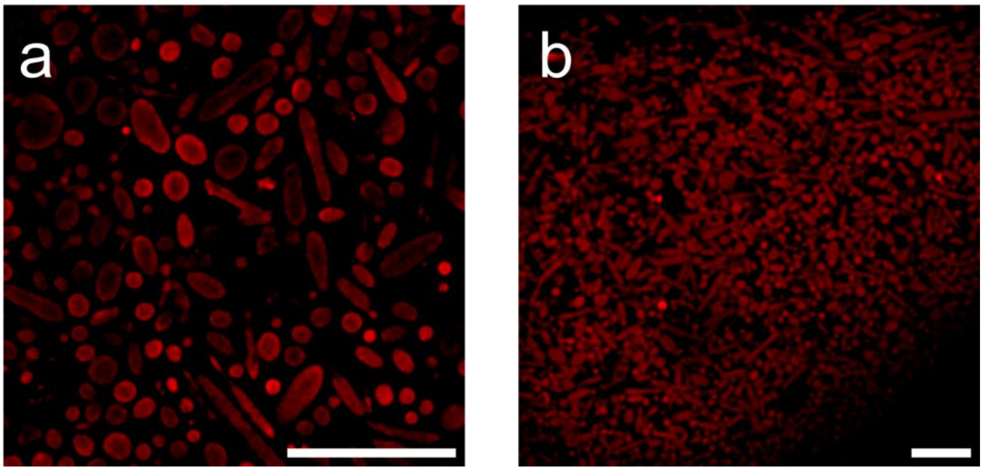

Figure S 3 CLSM micrographs of mixed vesicles after a short centrifugation at 50 g. a) High magnification, b) low magnification. Red: CPC. Scale bars: 100 µm.

Table S 1 Characterization of vesicles via area and aspect ratio, from the original mix and from simple centrifugation.

	Original mix	Simple centrifugation bottom	Simple centrifugation middle	Simple centrifugation top
**Area (μm^2)**				
Median	17.22	30.99	68.58	13.49
Range	137314	46899	8161	3526
75% percentile	115.2	81.35	155.0	47.06
Mean	560.7	208.2	197.4	77.28
Std. Deviation	6304	2138	554.3	258.3
Coefficient of variation	1124%	1027%	280.9%	334.3%
Skewness	20.69	21.04	9.346	8.470
**Aspect ratio**				
Median	0.6912	0.7413	0.7070	0.6570
Mean	0.6370	0.7097	0.6711	0.6333
Std. Deviation	0.1667	0.1418	0.1789	0.1262
Coefficient of variation	26.18%	19.98%	26.66%	19.93%
Skewness	-0.4187	-0.5783	-0.6669	-0.2815

Table S 2 Characterization of vesicles via area and aspect ratio, from sucrose gradient centrifugation.

	Sucrose bottom	Sucrose 40%	Sucrose 30%	Sucrose 20%	Sucrose 10%	Sucrose 0%
**Area (μm^2)**						
Median	72.03	60.26	20.09	22.38	6.887	12.05
Range	72798	4120	568.8	3152	197.4	196.3
75% percentile	198.7	137.3	35.58	60.84	15.50	24.11
Mean	809.2	146.4	33.55	96.10	13.11	19.08
Std. Deviation	4742	345.5	46.81	282.3	19.68	22.72
Coefficient of variation	586.0%	236.1%	139.5%	293.8%	150.1%	119.1%
Skewness	12.52	7.106	5.894	6.906	4.435	3.462
**Aspect ratio**						
Median	0.7284	0.7445	0.7794	0.6572	0.6860	0.7070
Mean	0.6992	0.6731	0.7373	0.6513	0.6555	0.6904
Std. Deviation	0.1708	0.2131	0.1344	0.1338	0.1234	0.1145
Coefficient of variation	24.43%	31.66%	18.23%	20.55%	18.83%	16.58%
Skewness	-0.7391	-0.7892	-1.385	-0.2663	-0.3398	-0.4188

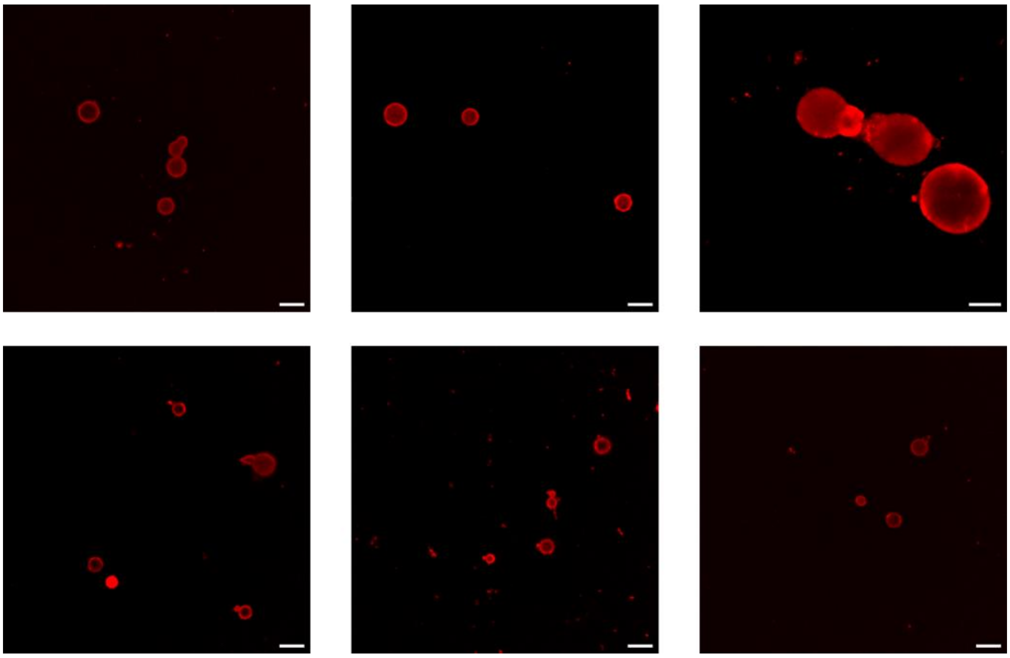

Figure S 4 CLSM micrographs of vesicles produced via slow stirring. Red: CPC. Scale bars: 10 µm.

Table S 3 Characterization of vesicles via area and aspect ratio, produced via slow stirring.

**Area (μm^2)**	
	Slow stirring
Median	11.84
Range	833.9
75% percentile	34.29
Mean	50.22
Std. Deviation	103.1
Coefficient of variation	205.2%
Skewness	3.939
**Aspect ratio**	
Median	0.6993
Mean	0.6915
Std. Deviation	0.1334
Coefficient of variation	19.30%
Skewness	-0.2947

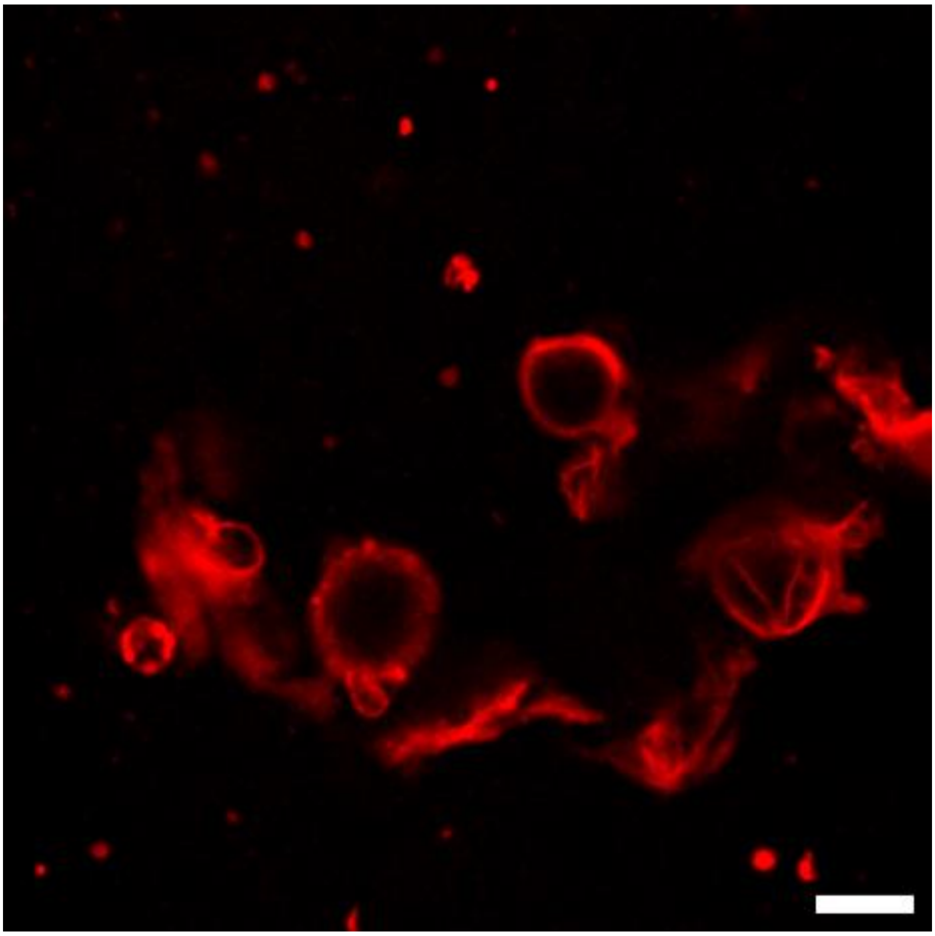

Figure S 5 CLSM micrograph of vesicles produced via fast stirring, showing the irregular shape of the vesicles. Red: CPC. Scale bar: 20 µm.

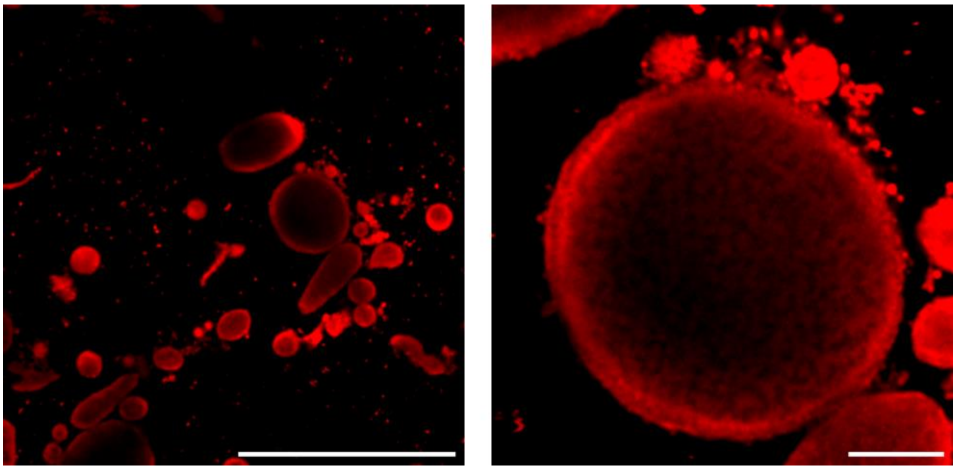

Figure S 6 CLSM micrographs of large vesicles (original mix, bottom fraction) highlighting the internal structures labelled by the probe. Red: CPC. Scale bars: 100 µm.

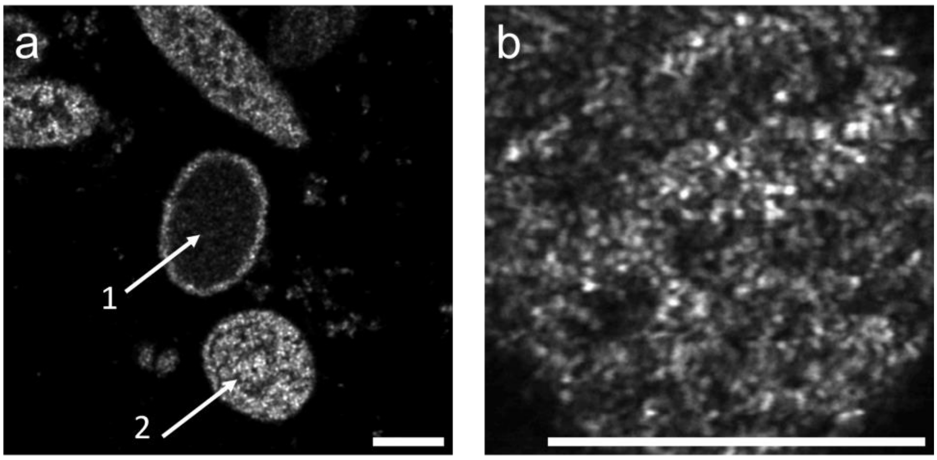

Figure S 7 a) CLSM micrograph of vesicles (original mix, bottom fraction) in reflection mode, highlighting the internal structures visible even without fluorescent probe. 1: “hollow” vesicle, 2: “filled” vesicle. b) close-up of vesicle 2. Scale bars: 50 µm.

Table S 4 Encapsulation into GVs with and without electroporation.

Group	Proportion of fluorescent GVs	Standard Error (SE)	P-value
Electroporated	0.8	0.10328	0.00729
Non-electroporated	0	0	

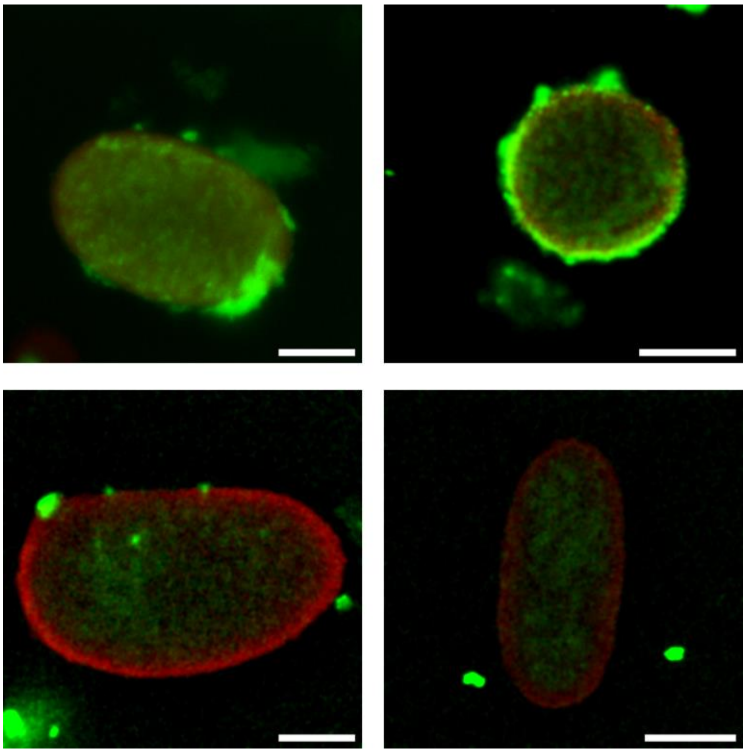

Figure S 8 CLSM micrographs of vesicles after electroporation in presence of eGFP. Some of the eGFP can be seen agglomerated outside of the vesicles. Green: eGFP, red: CPC. Scale bars: 20 µm.

Table S 5 Proportion of fluorescent vesicles depending on their internal structure.

Group	Proportion of fluorescent GVs	Standard Error (SE)	P-value
Filled	0.769	0.1168	0.203
Hollow	0.5	0.1176	

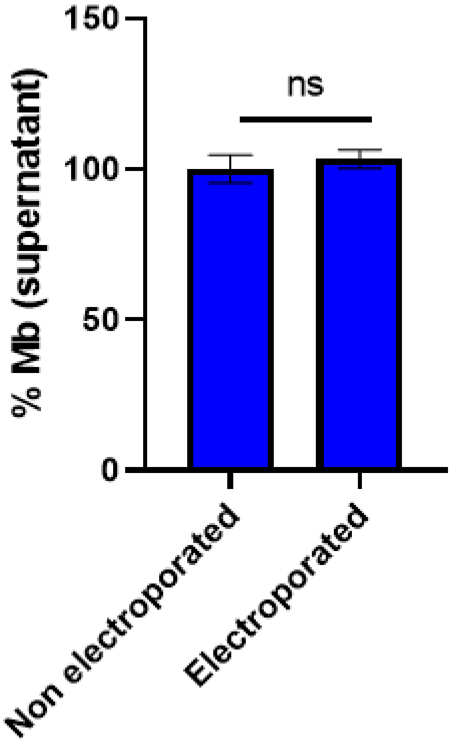

Figure S 9 Mb detected in the GV supernatant, before and after electroporation, showing no detectable release of the protein upon electroporation. N=3 replicates, ±SD, t-test. P>0.5.

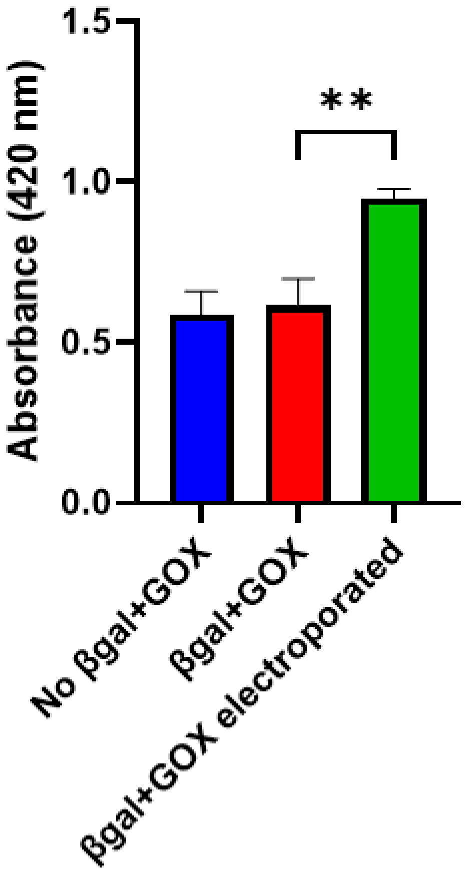

Figure S 10 TMB assay showing activity levels for the βgal-GOX-MB cascade in vesicles. Only the presence of the two electroporated enzymes leads to the oxidation of TMB, producing a product detectable at 420 nm. N=3 replicates, ±SD (one-way ANOVA).**:$p<0.01$.
